# The Prognostic Impact of Gender, Therapeutic Strategies, Molecular Background, and Tumor-Infiltrating Lymphocytes in Glioblastoma: A Still Unsolved Jigsaw

**DOI:** 10.3390/genes14020501

**Published:** 2023-02-15

**Authors:** Lorenzo Innocenti, Valerio Ortenzi, Rosa Scarpitta, Nicola Montemurro, Francesco Pasqualetti, Roberta Asseri, Stefano Lazzi, Anna Szumera-Cieckiewicz, Katia De Ieso, Paolo Perrini, Antonio Giuseppe Naccarato, Cristian Scatena, Giuseppe Nicolò Fanelli

**Affiliations:** 1 Division of Pathology, Department of Translational Research and New Technologies in Medicine and Surgery, University of Pisa, 56126 Pisa, Italy; 2 Department of Laboratory Medicine, Pisa University Hospital, 56126 Pisa, Italy; 3 Department of Neurosurgery, Pisa University Hospital, 56126 Pisa, Italy; 4 Department of Radiation Oncology, Pisa University Hospital, 56126 Pisa, Italy; 5 Department of Oncology, Oxford University, Oxford OX1 4BH, UK; 6 Anatomic Pathology Unit, Department of Medical Biotechnology, University of Siena, 53100 Siena, Italy; 7 Department of Pathology, Maria Sklodowska-Curie National Research Institute of Oncology, 02-781 Warsaw, Poland; 8 Department of Diagnostic Hematology, Institute of Hematology and Transfusion Medicine, 02-776 Warsaw, Poland; 9 Department of Pathology and Laboratory Medicine, Weill Cornell Medicine, New York, NY 10021, USA

**Keywords:** glioblastoma, microenvironment, gender, TILs, CD4, CD8, prognosis

## Abstract

Despite the adoption of novel therapeutical approaches, the outcomes for glioblastoma (GBM) patients remain poor. In the present study, we investigated the prognostic impact of several clinico-pathological and molecular features as well as the role of the cellular immune response in a series of 59 GBM. CD4+ and CD8+ tumor-infiltrating lymphocytes (TILs) were digitally assessed on tissue microarray cores and their prognostic role was investigated. Moreover, the impact of other clinico-pathological features was evaluated. The number of CD4+ and CD8+ is higher in GBM tissue compared to normal brain tissue (*p* < 0.0001 and *p* = 0.0005 respectively). A positive correlation between CD4+ and CD8+ in GBM is present (*r_s_ =* 0.417—*p* = 0.001). CD4+ TILs are inversely related to overall survival (OS) (HR = 1.79, 95% CI 1.1–3.1, *p =* 0.035). The presence of low CD4+ TILs combined with low CD8+ TILs is an independent predictor of longer OS (HR 0.38, 95% CI 0.18–0.79, *p =* 0.014). Female sex is independently related to longer OS (HR 0.42, 95% CI 0.22–0.77, *p =* 0.006). Adjuvant treatment, methylguanine methyltransferase (*MGMT*) promoter methylation, and age remain important prognostic factors but are influenced by other features. Adaptive cell-mediated immunity can affect the outcomes of GBM patients. Further studies are needed to elucidate the commitment of the CD4+ cells and the effects of different TILs subpopulations in GBM.

## 1. Introduction

Glioblastoma (GBM) is the most common and deadly primary malignant brain tumor of adults, accounting for approximately 70% of gliomas [1]. Despite the adoption of novel therapeutical approaches, outcomes remain poor with a 5-year survival of 5.5% [1,2].

GBM is a heterogeneous malignancy from both morphological and molecular viewpoints. The tumor mass is composed of several cancer and stromal cell types that interact with each other modulating tumor initiation, progression, and treatment response [3]. So far four GBM molecular subtypes have been identified with different aggressiveness, progression potential, and prognosis [4]. However, as extensively demonstrated, several other clinicopathological features, such as age, *MGMT* promoter methylation, and the extent of surgical resection (EOR) impact patient outcomes and therapy responses [5,6,7,8,9,10,11,12].

Nowadays, the complex interaction between GBM and its multifaceted microenvironment, and the prognostic impact of the latter remain poorly understood. Indeed, the so-called GBM immune microenvironment accounts for a significant tumor volume and appears to be composed of either resident or recruited immune cells; however, its different subpopulations and their biological role have been only partially characterized [1].

Under physiological conditions, the blood-brain barrier (BBB) plays a central role by maintaining an immunosuppressive milieu and limiting the extravasation of inflammatory cells. This fine homeostatic mechanism is altered during tumor progression, leading to the disruption of the normal BBB function [13,14]. Despite the presence of different lymphocytic subpopulations composing the GBM-associated tumor-infiltrating lymphocytes (TILs) [3], GBM is characterized by a markedly immunosuppressive status [15]. This could be responsible for reduced immune-mediated cancer cell death.

Contrasting data about TILs’ prognostic role in patients with high-grade gliomas are present in the literature. According to Sadfari et al. [16], an increased number of TILs correlated with poorer prognosis, whereas other authors [17,18] found a positive correlation between TILs subpopulation and patients’ prognosis, and further studies [19] failed to find any significant association.

Hence, shedding light on glioma-associated immune mechanisms could play a crucial role in the GBM patient’s management and could ultimately pave the way for the development of novel therapeutic approaches.

In the current study, we investigated the prognostic impact of different clinicopathological and molecular features as well as the role of the cellular immune response on patient outcomes in a well-annotated and molecularly characterized series of GBM.

## 2. Materials and Methods

### 2.1. Study Population

In the present study, 59 GBM adult patients were retrospectively selected from the University Hospital of Pisa. All patients underwent surgical resection from 2013 to 2020. None of them suffered from concomitant unrelated comorbidities, such as other neoplasms or infections. No chemo-radiotherapy was administered before the diagnosis of glioblastoma.

All cases were independently re-evaluated by two expert neuropathologists (V.O. and G.N.F) and discrepancies were solved by collegial discussion. Diagnostic criteria were based on the 2022 WHO Classification of CNS tumors.

EOR was classified into gross tumor resection (GTR) and subtotal tumor resection (STR), based on the extent of surgical radicality.

From the medical records, the administration of adjuvant radio- and chemotherapy, recurrence, and cancer-related death times were annotated. The last follow-up was performed in December 2022. Progression-free survival (PFS) was defined as the time from surgery to the time of the first recurrence. Overall survival (OS) was defined as the time from the primary diagnosis to the patient’s cancer-related death. Alive patients were censored at the date of the last follow-up. The median follow-up was calculated as suggested by Schemper et al. [20].

### 2.2. Tissue Microarray

There were two expert neuropathologists (G.N.F. and V.O.) that independently reviewed each case, selecting at least 2–3 regions of interest (ROIs) from GBM and 1–2 ROIs from normal-appearing tissue placed at a sufficient distance from the tumor, when it was available. Tissue cores (1.5 mm in diameter) were punched out from ROIs via the TMA Grand Master (3DHISTECH, Budapest, Hungary). Each tissue core was then embedded in the recipient paraffin blocks and registered according to the grid that was designed with the TMA Control software. In each TMA block, liver, tonsil, kidney, testis, and thyroid tissue were used as controls. Finally, 178 tissue cores of primitive GBM and 40 tissue cores of normal-appearing tissue were collected in 4 TMA blocks.

### 2.3. Immunohistochemistry and Imaging Analysis

Immunohistochemical (IHC) analyses were carried out for CD4, CD8, and p53 markers via Ventana Benchmark Ultra (Ventana Medical System—Roche). Briefly, 4 μm-lick FFPE sections were immunostained with CD4 antibody (SP35, rabbit monoclonal); CD8 antibody (SP57, rabbit monoclonal), and p53 (DO-7 mouse monoclonal), and developed in diaminobenzidine (DAB)–hydrogen peroxide for 10 min (ultraView Universal DAB kit, Ventana Medical System—Roche). Finally, sections were counterstained with hematoxylin and mounted. Positive controls were included for the current analysis (tonsil, testis, liver, and kidney).

Slide scanning was performed via Ventana DP 200 Slide Scanner (Roche Diagnostics International, Rotkreuz, Switzerland). Imaging analyses were carried out through QuPath (v0.3.0, Belfast, XI) platform; a semiautomatic approach was applied and the number of total positive cells for each core and the average number of positive cells per high power field (HPF) were assessed. *TP53* was considered mutated if no expression or a strong and diffuse expression was present in GBM cells.

### 2.4. Molecular Characterization

A total of two 10 μm-thick sections were obtained from each FFPE specimen, and DNA was extracted according to NucleoSPin Tissue protocol (Genomic DNA from Tissue-Macherey-Nagel Gmbh and Co.Kg., Duren, Germany). Negative controls were included in the present analysis. DNA quantification was performed via Qubit 2.0 (Life Technologies, Carlsbad, CA, USA) and was in a range of 50–500 ng/μL.

The analyses for IDH1 (codon 132) and IDH2 (codon 172) were carried out using Diatech Pharmacogenetics IDH1/2 status kit. Following DNA polymerase amplification, all samples were pyrosequenced (PyroMark Q96ID, Quiagen, Redwood, CA, USA) and analyzed by ProMark ID platform.

For the detection of the chromosomal 1p/19q co-deletion, MLPA was performed using the SALSA MLPA kit and P088 probe mix (MRC Holland, Amsterdam, the Netherlands) according to the manufacturer’s protocol. Samples were analyzed on a 3500 DNA sequencer (Applied Biosystems, Waltham, MA, USA).

The methylation profile of the *MGMT* promoter was assessed using MGMT plus kit (Diatech Pharmacogenetics). DNA was treated with bisulfite and amplified using specific primers (Takara Ex Taq DNA polymerase—Clonech). Pyrosequencing was carried out and elaborated via the Pyro Q-CpG platform. The cut/off value was set to 7%.

### 2.5. Statistical Analyses

Categorical variables were compared using a Chi-Square test and Fisher’s exact test, whereas quantitative and ordinal variables were compared using a Mann–Whitney U test and Kruskal–Wallis test (with Dunn test for multiple comparisons). A Spearman rho test was used to assess the relationship between biomarkers. *MGMT* promoter methylation status and TILs levels have been dichotomized (cut-off values are defined above and below). Kaplan–Meier curves were used to estimate survival outcomes; the log-rank test was used to compare different groups. Cox proportional hazard models were used to calculate hazard ratios (HRs) of recurrence or death according to the number of CD4, and CD8+ TILs in GBMs.

All analyses were performed with SPSS 26.0 (SPSS Inc., Chicago, IL, USA), and graphs were drawn using GraphPad Prism (GraphPad Software, San Diego, CA version 9.1.1). Results were classified as statistically significant if their *p*-values were <0.05.

## 3. Results

### 3.1. Clinicopathological Findings

Among the 59 GBM patients, 34 (58%) were male and 25 (42%) were female. The mean age at diagnosis was 62.15 ± 10.9 years (median 62; range 26–80 years). The GBM site was quite heterogeneous: 12 (20%) GBMs were localized in the frontal lobe, 11(19%) were in the parietal lobe, 9 (15%) in the temporal lobe, 1 (2%) in the occipital lobe, and 1 (3%) was localized at the insula. Some GBM involved a different lobe namely 8 (13.5%) the fronto-parietal area, 5 (8%) the fronto-temporal area, 8 (13.5%) the temporo-parietal area, 2 (3%) the parieto-occipital area, and 2 (3%) the temporo-occipital area.

The EOR was dichotomized as gross total resection (GTR), reached in 24 (40%) patients, and subtotal resection (STR) was obtained in 35 (60%) patients.

In 10 GBM (17%), 1p/19q co-deletion was found. A total of 42 (71%) GBM showed *MGMT* promoter methylation, whereas only 3 (5%) harbored a mutation at codon 132 of the *IDH1* gene. No GBMs had *IDH2* mutations. *TP53* was mutated in 8 (13.5%) GBM. The mean proliferation index (ki-67) was 33.4% (median 35%; range 10–90%).

Radiotherapy alone was administered in 16 (27%) patients, chemo-radiotherapy was administered in 18 (31%) patients, whereas 25 patients did not receive any treatment after surgery.

A total of 23 (39%) patients recurred after the first surgery and 54 (92%) died during the follow-up. The median follow-up was 58.67 months (95% CI 37.52–78.82; range 1.03–82.2). The median PFS was 10.97 months (95% CI 6.51–15.43; range 1.03–78.8 months) and the median OS was 18.87 months (95% CI 15.3–22.44; range 1.03–82.2).

**Figure 1:**
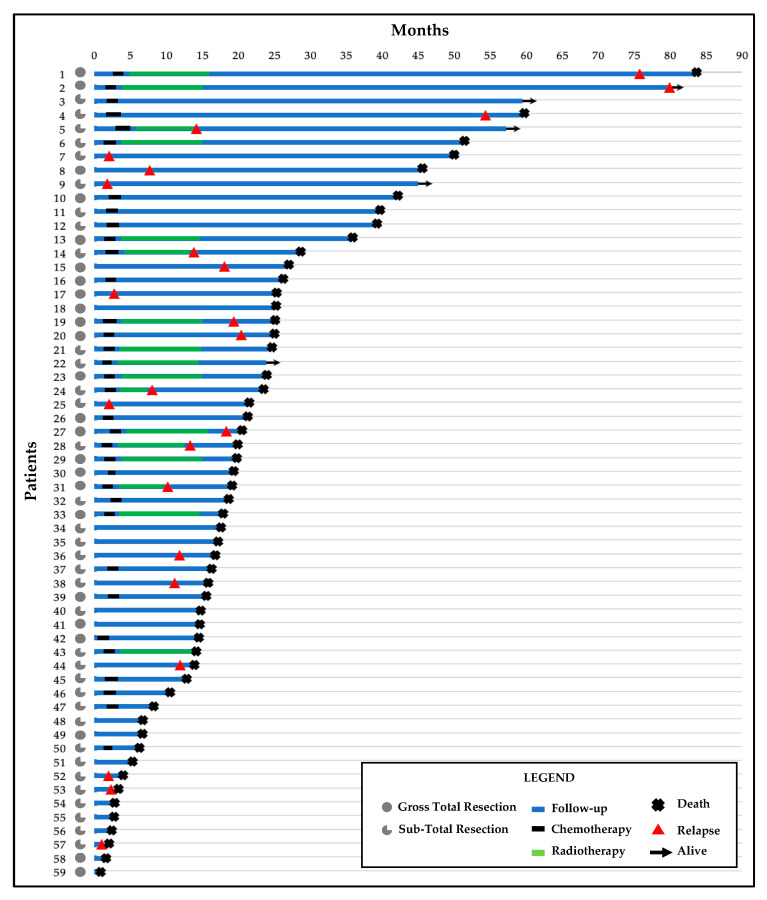
Swimmer’s plot with follow-up time, treatments, and clinical outcomes for the 59 patients.

All clinicopathological findings are summarized in Table 1 and Figure 1.

### 3.2. Clinico-Pathological Features and Prognosis

In our cohort, age was not related either to OS or PFS in univariate analysis but was related to both in multivariate analysis (see below).

**Figure 2:**
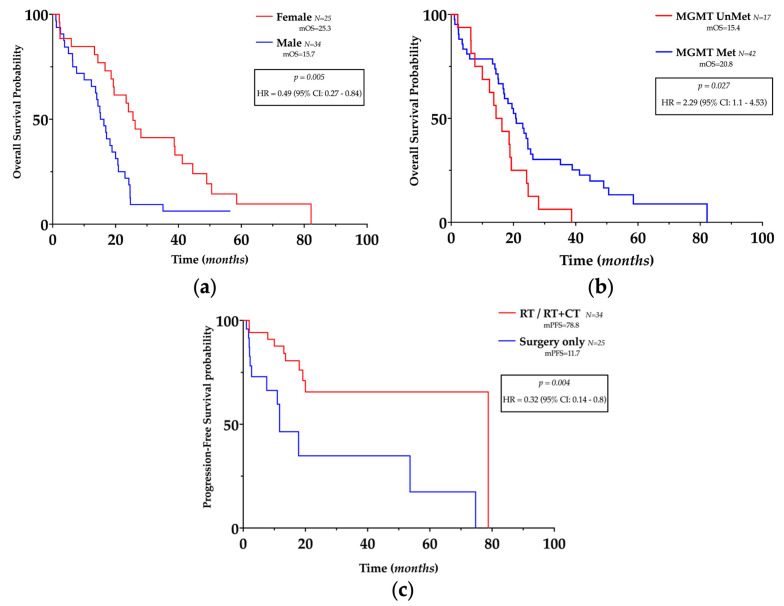
Prognostic impact of clinicopathological features in glioblastoma. (**a**,**b**) Kaplan–Meier plots of overall survival, according to gender and *MGMT* promoter methylation status. (**c**) Kaplan–Meier plots of progression-free survival according to treatment.

Interestingly, females had a longer OS (HR 0.49, 95% CI 0.27–0.84, *p = 0.005*) (Figure 2a) in univariate analysis, and gender was related to OS and PFS in multivariate analysis (see below).

Despite GTR being related to a longer OS (GTR: mOS = 23.4 vs. STR: mOS = 15.8) and PFS (GTR mPFS = 18.9 vs. mPFS = 10.5), no statistical significance was reached either in univariate or multivariate analyses.

*MGMT* promoter methylation was related to longer OS (HR = 2.29, 95% CI 1.1–4.53, *p = 0.027*) (Figure 2b) in univariate analysis but not in multivariate analysis and was not related to PFS.

Different mOS were registered according to the treatment approach (surgery only: mOS = 14.4, RT: mOS = 19.5, RT + CT: mOS = 23.19) as well as different mPFS (surgery only: mPFS = 11.7, RT: mPFS = 19.1, RT + CT: mPFS = 78.8); however, no statistically significant superiority in efficacy was found between treatments. However, as expected, post-surgery medical treatment (regardless of the approach) was related to longer PFS (HR 0.32, 95% CI 0.14–0.8 *p =* 0.004) (Figure 2c) both in univariate and multivariate analysss (see below), but unexpectedly, not to OS.

Finally, the proliferation index, 1p/19q co-deletion, *TP53,* and *IDH1* status were not related to OS and/or PFS.

### 3.3. TILs Characterization in GBM and Normal Tissue

**Figure 3:**
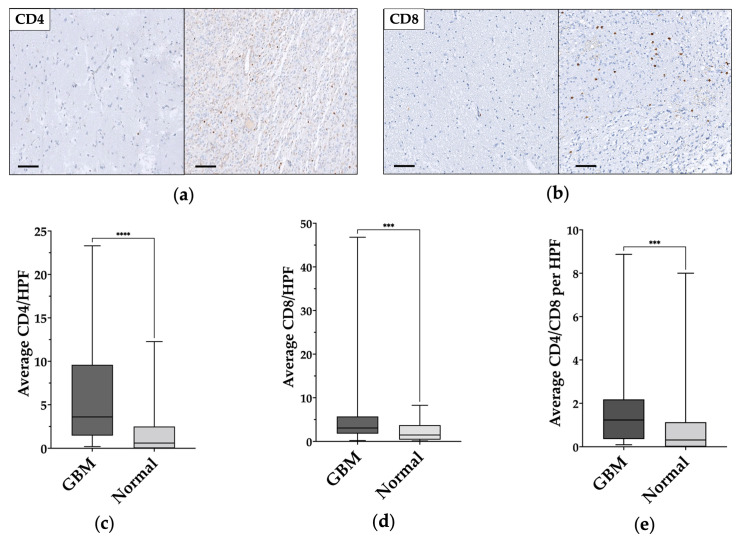
CD4+ and CD8+ cells comparison in normal-appearing tissue and in glioblastoma samples. (**a**,**b**) Immunohistochemical staining shows the low levels/absence of CD4+ and CD8+ in normal-appearing tissue on the left and tumor-infiltrating lymphocytes (TILs) in glioblastoma on the right. Magnification of all images 200×. Scale bar: 100μm. (**c**–**e**) Statistically significant CD4+, CD8+, and CD4/CD8 differences between normal-appearing tissue and glioblastoma (GBM) samples. ***: *p* ≤ 0.001; ****: *p* ≤ 0.0001.

TILs populations were characterized according to the expression of CD4 or CD8 (Figure 3a,b). The total number of each population was digitally assessed and normalized according to the number of positive cells in each HPF, the mean and median values were then calculated for each core, and finally for each patient.

In normal-appearing tissue samples, the average number of CD4+ and CD8+ cells per HPF were 1.78 ± 2.7 (median 0.6) and 2.28 ± 2.17 (median 1.47), respectively; whereas, in GBM samples the average number of CD4+ and CD8+ cells per HPS were 6.18 ± 6 (median 3.64) and 6.14 ± 8.91 (median 3.07), respectively. CD4/CD8 ratio was 1.26 ± 0.3 (median 0.31) in the normal-appearing samples and 1.86 ± 2.09 (median 1.23) in the GBM samples.

A statistically significant difference was found between normal-appearing and GBM samples for CD4+ cells (*p <* 0.0001) (Figure 2c) and CD8+ cells (*p* = 0.0005) (Figure 3d), and for CD4/CD8 ratio (*p =* 0.0009) (Figure 3e). The data and results are summarized in Table 2.

No differences were found between the number of CD4+ and CD8+ cells within GBM samples or within normal-appearing tissue. However, we found a moderate positive correlation between CD4+ and CD8+ number of cells in GBM (*r_s_ = 0.417—p =* 0.001) but not in normal-appearing tissue.

The number of CD4+ and CD8+ TILs or their CD4/CD8 ratio did not vary significantly according to age, sex, tumor site, surgery type, relapse, *MGMT* promoter methylation, 1p/19q co-deletion, *IDH1*, and *TP53* mutations.

### 3.4. TILs Levels and Clinical Outcomes

**Figure 4:**
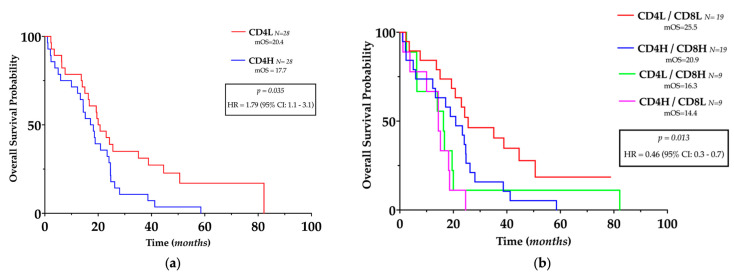
Prognostic impact of TILs in glioblastoma. (**a**) Kaplan–Meier plots of overall survival, stratified by the predefined cut-off points for CD4+ cells. (**b**) Kaplan–Meier plots of overall survival, stratified according to the different TILs pattern stratified by the predefined cutoff points for CD4+ and CD8+ cells. L: low; H: High.

For survival analyses, the median number of CD4+ and CD8+ TILs per HPF was used as the cut-off point to define the high (H) and low (L) infiltrated GBM samples, as described by previous authors [17]. The number of CD4+ cells was inversely related to OS (HR = 1.79, 95% CI 1.1–3.1, *p* = *0.035*) (Figure 4a) but not to PFS, whereas the number of CD8+ cells or CD4/CD8 ratio was not related to OS or PFS.

Since a positive correlation between CD4+ and CD8+ cells was present in GBM, we investigated their complementary role in patients’ outcomes; hence, GBM patients have been divided into four different subgroups according to the number of CD4+ and CD8+ cells as follows: CD4H/CD8H, CD4H/CD8L, CD4L/CD8H, and CD4L/CD8L. Compared with all the other groups, patients with low CD4+ with concurrent low CD8+ had significantly longer OS (HR = 0.46, 95% CI = 0.3–0.9, *p = 0.0013*) (Figure 4b) but not longer PFS.

Cox regression analyses confirmed that low CD4+ combined with low CD8+ was an independent predictor of longer OS (HR 0.38, 95% CI 0.18–0.79, *p = 0.014*) (Table 3) but was not related to PFS.

## 4. Discussion

During the last decade, the survival outcome of GBM patients has improved thanks to the introduction of the Stupp protocol [7], which provides a multimodal treatment based on surgery and post-operative administration of chemo-radiotherapy. However, the prognosis remains poor in the majority of cases. Hence, novel approaches, including immunotherapy, and novel prognostic stratification based on different clinicopathological, and molecular features are currently being thoroughly investigated. As extensively demonstrated for other malignancies [21,22,23,24,25,26,27,28], the efficacy of tailored therapy such as immunotherapy relies on different specific cancer features assessed in serum [29,30,31] or directly on tissue, such as TILs [32,33].

In the present study, we have investigated the prognostic role of several clinicopathological features and how CD4+ and CD8+ TILs can impact patients’ outcomes in a well-annotated and molecularly characterized series of GBM.

Interestingly, as already demonstrated in other larger cohorts [10,34], gender plays an important prognostic role in GBM: female patients show longer survival than males. In orthotopic GBM models, Barone et al. [35] demonstrated that high estrogen levels increase survival, and Li et al. [36] observed a high frequency of estrogen receptor methylation in a series of GBM. In contrast, Yu et al. [37] found how androgen receptor signaling can promote GBM cancerogenesis in adult men by the inhibition of the TGF-β receptor. Moreover, Khan et al. [9] demonstrated in an in-silico analysis several crucial differences in immune system and Wnt pathway between gliomas from male and female patients. All these findings support our results and demonstrate how hormone-based therapy could represent a novel therapeutic approach.

Age remains an important prognostic factor in GBM patients. Indeed, its incidence changes with age [38], and despite the same histological features displayed, outcomes can vary significantly by age [39,40]. However, Jia et al. [41] recently demonstrated how age is not non-linearly related to prognosis, challenging the applicability of current age subgroupings and highlighting the unmet need for individualized treatment guided by age. This is in line with our data that pinpoint how age is not an independent prognostic factor but is influenced by the treatment approach and other important clinical and molecular variables such as gender, surgical resection, *MGMT* promoter methylation status, and TILs subpopulations.

The EOR for GBM has been a matter of debate for decades. Although several studies [42,43,44,45] and meta-analyses [42] support the superior efficacy of GTR on survival and tumor progression, the concept of “maximal safe resection” (MSR) should be considered [46]. MSR is the maximal, safely achievable volumetric resection, and should be reached by the additional removal of the FLAIR abnormal regions, when safely feasible; this may lead to longer survival without significant increases in neurological postoperative morbidity [47]. However, the definition of MSR should become distinctive for each patient, and in general, surgical strategies, as for other therapeutic approaches, should be chosen after the GBM molecular characterization and tailored to the patient’s clinical status [48,49,50,51]. In our cohort, even if we registered better outcomes in GTR subgroup, we did not reach the significance for both OS and PFS; this could be related to the small sample size or to a selection bias; or else it could partially reflect the complexity of the GBM in which the surgery is only a part of the multimodal therapeutic approach, and its efficacy may be influenced by other elements.

The current standard of medical treatment after MSR is the concurrent administration of Temozolomide (TMZ), an alkylating agent, and RT, followed by six cycles of adjuvant TMZ [6,52]. However, these approaches must be modulated based on the patient’s clinical status and the molecular landscape of the GBM. The most cytotoxic effect that is induced by TMZ is the alkylation of the O^6^ position of guanine, which is reverted by the DNA repair protein *MGMT* [8]. The aberrant methylation of the *MGMT* promoter region results in gene silencing, decreasing the ability to repair DNA damage that is induced by chemotherapy. In the pivotal trial of Stupp et al. [7], *MGMT* promoter methylation was strongly associated with longer OS in the experimental arm (TMZ + RT) but had only a minor prognostic impact for PFS in patients receiving RT alone [5], suggesting its predictive role. Indeed, this assessment has been integrated into the routine GBM molecular characterization. Our data confirmed this assumption where *MGMT* promoter methylation was related to a longer OS but not to PFS. Finally, in multivariate analysis, we found a correlation between medical treatment (RT or RT + CT) and PFS but not for OS. These discrepancies with the literature could be related to our small and quite heterogeneous sample size.

Although the brain is an immune-privileged tissue in which adaptive immunity and inflammation are highly controlled, several authors have documented lymphocytic infiltration into large series of gliomas [53,54]. However, whether these TILs aid cancer immuno-suppression [55,56] or contribute to cancer cells immune-mediated death [57] is largely unknown. TILs assessment and their interplay with other clinicopathological and molecular features may partly uncover their role in GBM patients’ prognosis.

In the present study, we assessed the number of CD4+ and CD8+ TILs to evaluate the prognostic role of adaptive cell-mediated immunity in GBM. We showed how the average number of these specific TILs is increased in GBM samples in comparison to normal-appearing tissue. However, no significant differences between the number of CD4+ and CD8+ were found within GBM samples or within normal-appearing tissue. Conversely, we found a positive correlation between the two TILs populations in GBM samples. This could be related to a partial or complete disruption of the BBB and to increased angiogenetic processes that lead to uncontrolled extravasation of different TILs subpopulations [58]. Alternatively, GBM cells can retrieve TILs from the bloodstream. Indeed, Rutledge et al. [3], found a positive correlation between TILs and morphologic features, molecular GBM subclasses, and mutational profiles, but no relation with outcomes was confirmed. Nevertheless, in general, the total number of TILs in GBM is lower than in other tumors [18,32] and this could be related to the local microenvironment, which deserves further research.

As a result of a broad range of mechanisms, including senescence, tolerance, anergy, and exhaustion, GBM often induces a state of global T-cell dysfunction and active immunosuppression, through an increased expression of inhibitory receptors including CTLA-4, CD73, and CD39, which results in a reduction in T-cell activity [59]. Waziri et al. [60] confirmed these data, demonstrating how the majority of CD4+ TILs in GBM inhibit the cellular immune reaction, hampering the CD8+ cytotoxic function. Therefore, despite an increase in total CD4+ TILs, the immune function of GBM patients may be impaired. Indeed, in our cohort, high levels of CD4+ TILs were predictive of lower OS.

Previous studies reported how CD8+ TILs are positively related to outcomes in other malignancies [18,32,61,62], and recently Mauldin et al. [63] confirmed this assumption in GBM. However, in the present study, in line with the results of Kim et al. [64], we suggested that the number of CD8+ TILs or CD4/CD8 ratio cannot alone predict patient outcomes. Moreover, Mauldin et al. [63], found a lower density of CD4+ than that of CD8+, which is in contrast with our findings, but this may reflect the complex intra- and inter-tumor heterogeneity, and further study with larger multicentric cohorts can overcome these discrepancies.

Han et al. [17] described a positive trend in the raw numbers for several TILs populations from low-grade to high-grade gliomas; we confirmed these data revealing higher levels of CD4+ and CD8+ in the GBM samples. This could suggest their pivotal role in glioma progression and corroborates the hypothesis that numerous interactions within TILs subpopulations may occur in GBM carcinogenesis, prompting further research. Hence, we examined the prognostic role of different patterns of CD4+ and CD8+ TILs demonstrating how low levels of CD4+ combined with low levels of CD8 represent an independent positive prognostic factor. Additionally, we partially confirmed previous studies [17] in which high CD4+ cells combined with low CD8+ cells represent the TILs pattern with the least favorable OS, even if this comparison was not statistically significant in our cohort.

The prognostic role of these different TILs patterns supports the hypothesis of unbalanced activation of the cellular immune response in GBM, which could hamper current treatment strategies. Low CD4+ combined with low CD8+ may suggest a lesser but effective and balanced anti-cancer immune activation that could lead to a better OS; in contrast, high CD4+ combined with low CD8+ TIL levels may be related to a suppressed cellular immune response and consequently to worse outcomes. Indeed, Perrin et al. [65] reported that even with an adequate number of CD8+ TILs, the immune system is not able to prevent GBM growth; this could be related to a deficient CD4+ helper activation.

It is well known that CD4+ TILs could display a double immune activity in human cancers. On one hand, CD4+ T-helpers play a central role in the activation, recruitment, and modulation of several aspects of the adaptive immune response. Conversely, CD4+ Tregs can reduce anti-tumor immunity and accelerate its progression [66,67]. In fact, without sufficient CD4+ T-helper support, CD8+ T-cells usually cannot perform their full potential in vivo [68,69]. Recently, Mitsdoerffer et al. [70] found that the transcriptome of CD8+ TILs in GBM was coherent with a strong anti-tumor response, while that one of CD4+ TILs showed a strong commitment to the Th17 differentiation that may negatively modulate the anti-tumor immune response.

Finally, in the present study, we did not find significant differences in TILs subpopulation according to age, tumor site, EOR, *MGMT* promoter methylation, proliferation index, 1p/19q c-deletion, *TP53*, *IDH1/2* status, and risk of recurrence, which suggests that these prognostic factors may have an impact on patient prognosis in different ways.

## 5. Conclusions

In the present study, we underline the inherent complexity of the relationship between the immune system and GBM. Several clinicopathological and molecular features concur with the patients’ outcomes and must be considered in the therapeutic setting. We confirmed the prognostic impact of *MGMT* promoter methylation and adjuvant treatment. Interestingly, gender also played an important role in overall survival. Finally, we demonstrated how adaptive cell-mediated immunity can affect the outcomes of GBM patients: low CD4+ TILs levels alone or in combination with low CD8+ TILs were associated with better prognosis. Further studies are needed to better elucidate the commitment of the CD4+ cells and the effects of different TILs subpopulations in GBM. New insights derived from these efforts could lay the foundation for future target immunotherapy.

## Figures and Tables

**Table 1:** Clinicopathological data.

Clinicopathological Data	
Number of Patients	59
Mean Age *(range)*	62.15 y (26–80)
Gender	M = 34 (58%)
	F = 25 (42%)
Tumor site	Frontal = 12 (20%)
	Parietal = 11 (19%)
	Temporal = 9 (15%)
	Occipital = 1 (2%)
	Fronto-parietal = 8 (13.5%)
	Fronto-temporal = 5 (8%)
	Temporo-parietal = 8 (13.5%)
	Parieto-occipital = 2 (3%)
	Temporo-occipital = 2 (3%)
	Insular 1 (3%)
Extent of Surgical Resection	GTR = 24 (40%)
	STR = 35 (60%)
*MGMT* promoter	Met = 42 (71%)
	Unmet = 17 (29%)
*IDH1*	Mut = 3 (5%)
	WT = 56 (95%)
*IDH2*	Mut = 0 (0%)
	WT = 59 (100%)
1p/19q co-deletion	Y = 10 (17%)
	N = 49 (83%)
Mean Proliferation Index (ki-67—*range*)	33,4% (10–90%)
*TP53*	Mut = 8 (13.5%)
	WT = 51 (86.5%)
Treatment	RT only = 16 (27%)
	RT + CT = 18 (31%)
	N = 25 (42%)
Recurrence	Y = 23 (39%)
	N = 36 (61%)
Death	Y = 54 (92%)
	N = 5 (8%)
mPFS (*months*) *(95% CI; range)*	10.97 (6.51–15.43; 1.03–78.8)
mOS (*months*) *(95% CI; range)*	18.87(15.3–22.44; 1.03 –82.2)
mFollow-up (*months*) *(95% CI; range)*	78.7 (48.97–108.57; 1.03–82.2)

**Table 2:** Schematic representation of the TILs distribution in glioblastoma (GBM) and normal-appearing tissue.

TILs Distribution			
	GBM	Normal-Appearing Tissue	*p*
Mean CD4/HPF	6.18 ± 6 (median 3.64)	1.78 ± 2.7 (median 0.6)	<0.0001
Mean CD8/HPF	6.14 ± 8.91 (median 3.07)	2.28 ± 2.17 (median 1.47)	0.0005
Mean CD4/CD8	1.86 ± 2.09 (median 1.23)	1.26 ± 0.3 (median 0.31)	0.0009

**Table 3:** Cox regression analyses of different parameters for overall survival and progress-free survival. EOR: extent of resection; Met: Methylation; RT: radiotherapy; CT: Chemotherapy.

Multivariate Analysis						
	Overall Survival			Progression-Free Survival		
VARIABLE	*p*	HR	95% CI	*p*	HR	95% CI
Gender	0.006	0.42	0.22–0.77	0.027	0.31	0.1–0.85
Age	0.019	2.15	1.15–4.12	0.048	0.37	0.13–0.97
EOR	0.129	1.58	0.88–2.88	0.141	0.46	0.15–1.24
*MGMT* Met	0.081	0.54	0.27–1.1	0.406	1.62	0.55–5.58
RT	0.767	0.77	0.42–1.84	0.004	0.07	0.01–0.35
RT + CT	0.769	0.77	0.42–1.9	0.038	5.61	1.28–39.04
CD4L/CD8L	0.014	0.38	0.18–0.79	0.495	1.43	0.44–5.26

## Data Availability

Not applicable.
